# Strategic delivery of omega-3 fatty acids for modulating inflammatory neurodegenerative diseases

**DOI:** 10.3389/fnagi.2025.1535094

**Published:** 2025-03-17

**Authors:** Yixin Chen, Roni Touboul, Yao Chen, Chuchun L. Chang

**Affiliations:** ^1^Institute of Human Nutrition, Columbia University Vagelos College of Physicians and Surgeons, New York, NY, United States; ^2^Division of Gastroenterology, Hepatology, and Nutrition, Department of Pediatrics, Columbia University Vagelos College of Physicians and Surgeons, New York, NY, United States

**Keywords:** Alzheimer’s disease, fatty acids, neuroinflammation, omega-3, 5xFAD, microglia

## Abstract

**Objectives:**

Early-life inflammatory events like infections and injuries may predispose the brain to Alzheimer’s disease (AD) by disrupting neurodevelopment and raising vulnerability. The association between early neuroinflammation and subsequent neurodegeneration leading to dementia remains unclear. We hypothesize that omega-3 (n-3) fatty acids (FA), especially eicosapentaenoic acid (EPA) and docosahexaenoic acid (DHA), positively regulate neuro-immune cells, preserving their cell membrane structure and metabolic homeostasis. Our study examined whether strategic delivery of n-3 FA via injectable n-3 triglycerides (TG) can influence microglial lipid metabolism to prevent or delay AD progression.

**Methods and results:**

We characterized n-3 treatment effects on modulating lipid and metabolic homeostasis in microglia during the critical window of brain development. Our preliminary studies on determining the effects of early n-3 treatment on brain cell homeostasis indicate that perinatal bolus n-3 TG injections suppressed activation of gliosis-associated markers in young mice predisposed to AD (5xFAD) and yielded sustained regulatory effects on the expression of inflammatory molecules, such as interleukin-6 (*Il6*) and tumor necrosis factor-alpha (*Tnfα*), in adult brains. A significant increase in high-frequency ultrasonic vocalizations (USV) was observed in P6 5xFAD mice that received perinatal n-3 compared to vehicle control, implicating enhanced active communication patterns. Improvement in behavior deficits was observed in n-3-treated adult AD mice. Perinatal n-3 TG treatment modified brain lipid composition in young offspring, increasing key membrane lipid species, such as phospholipids (PL) and lysophospholipids (lysoPL). Pro-inflammatory sphingolipids associated with neurodegeneration, including lactosylceramide, were significantly lower in mice treated with n-3 than those in saline-treated AD mice.

**Conclusion:**

Our study establishes a proof of principle for targeting brain immune cell metabolism with injectable n-3 TG to mitigate neuroinflammation in AD pathogenesis, paving the way for future research into early treatments for related central nervous system (CNS) disorders.

## 1 Introduction

Neuroinflammation is a prevalent feature in the brains of those with Alzheimer’s disease (AD) (Heneka et al., 2015; Sobue et al., 2023; Loving and Bruce, 2020; Bachiller et al., 2018), a disease that poses significant public health and economic challenges not only in America but also worldwide. The classical hallmarks of AD pathology include the formation of extracellular amyloid-beta (Aβ) plaques as well as neurofibrillary tangles and neuropil threads induced by aggregation of tau into insoluble fibrils (Gomez-Ramos and Moran, 1998; Azargoonjahromi, 2024). Familial AD arises from specific mutations. In contrast, sporadic AD, the more common variant, is linked to a complex interplay of chronic neuroinflammation and metabolic dysfunction. Genetic studies on AD risk have identified a considerable number of genes that play a pivotal role in immune responses; the expression of these genes is predominantly found in microglia or infiltrating macrophages (Sobue et al., 2023; Bis et al., 2020; Wightman et al., 2021).

A continuing buildup of toxic Aβ and tau proteins can lead to the hyperactivation of central nervous system (CNS) innate immune cells, particularly microglia (Gao and Hong, 2008). This leads to the over-production of pro-inflammatory cytokines, reactive oxidative species, and subsequent neuronal death. Sustained neuroinflammation compromises the integrity of the blood–brain barrier, which allows a greater influx of circulating immune cells, triggering a vicious cycle of damage within the CNS (Aktas et al., 2007). Nonetheless, microglia play a vital role in both brain development and maintaining homeostatic functions through processes like synaptic pruning and myelination (Domingues et al., 2016; Maitre et al., 2023; Michell-Robinson et al., 2015); their metabolism is catered to meet increased bioenergetic demands involving phagocytosis and cytokine production. Disease-associated microglia (DAM) that are found in an AD model have distinct transcriptional signatures with genes associated with lipid and lipoprotein metabolism (Loving and Bruce, 2020). These DAM-resemble microglia are present in regions exhibiting active brain cell growth and in several CNS disease models (Deczkowska et al., 2018). Persistent neuroinflammation impairs the homeostatic and metabolic functions of microglia; however, the exact link between the decline in microglial function and the extent of neurodegeneration remains unclear.

Omega-3 (n-3) fatty acids (FA), especially eicosapentaenoic acid (EPA) and docosahexaenoic acid (DHA), are major structural and bioactive components of cellular membranes that are crucial for preserving cell viability and tissue homeostasis during systemic inflammation (Chang and Deckelbaum, 2013; Siddiqui et al., 2007; Hou et al., 2016; Calder, 2012). Emerging evidence, including our prior reports, indicates that bioactive n-3 FAs modulate inflammatory pathways by altering membrane microstructures, influencing transcriptional regulation, and promoting the production of anti-inflammatory mediators (Chang and Deckelbaum, 2013; Calder, 2012; Serhan, 2009; Manual Kollareth et al., 2020; Serhan, 2010; Calder, 2006; Serhan, 2014; Li et al., 2013). DHA contributes a major portion of the membrane FA acyl chains in the brain (Stillwell et al., 2005), suggesting its critical role during brain development and repair following injury. Reduced DHA in AD brains has been linked to cognitive alterations (Conquer et al., 2000). DHA may be protective against AD through several mechanisms with some characteristics similar to the favorable regulation of n-3 FA on systemic inflammatory responses as described above. In several AD animal models, oral feeding of n-3-rich diet or supplements decreased expression or levels of pro-inflammatory tumor necrosis factor-alpha (*Tnfα*), interleukin-1 beta (*Il1β*), or glial fibrillary acidic protein (GFAP) (Labrousse et al., 2012). Yet, the benefits of chronic n-3 supplementation in human AD trials remain controversial (Burckhardt et al., 2016). Importantly, chronic oral intake of fatty fish or fish oil supplements does not promptly raise n-3 FA concentrations in plasma and cell membranes. Oral n-3 triglycerides (TG) administration incorporates into cell membranes over several days to weeks, exerting anti-inflammatory benefits (Arterburn et al., 2006). However, high levels of DHA are not further increased by additional dietary n-3 FA supplementation (Orr et al., 2013). Membrane enrichment can be markedly accelerated by infusion of an n-3 TG-rich particles (TGRP) lipid emulsion (Hu et al., 2021). Few studies have evaluated the anti-inflammatory action of n-3 DHA or EPA in CNS via bolus injections. This route represents a feasible tool to uncover therapeutic potential during the specific window of brain pathogenesis.

Unlike other AD pathologic hallmarks, neuro-inflammatory alteration can occur and be highly detrimental early in life, leading to outcomes such as death, encephalitis or cognitive impairment (Hagberg et al., 2015; Bauman et al., 2014). Early-life events, such as neonatal hypoxia-ischemia (H/I), have been shown to induce AD-associated Aβ deposits and tau protein dysfunction, which can be further exacerbated by the presence of inflammatory microglia and macrophages at injury sites (Pluta et al., 2020; Pluta et al., 2018; Ulamek-Koziol et al., 2017). Additionally, a history of multiple traumatic brain injuries at early ages has been linked to an increased AD risk (Van Den Heuvel et al., 2007), suggesting a potential link between brain insult in the earliest stages of life and the occurrence of long-lasting genetic and biochemical changes leading to AD-associated neurodegeneration. Notably, bolus IP injections of n-3 DHA offer neuroprotection in neonatal mice subjected to H/I injury in the brain (Williams et al., 2013; Mayurasakorn et al., 2016) and in adult models who underwent spinal cord injury (Liu et al., 2017; Paterniti et al., 2014). A recent study shows that maternal dietary n-3 FA deficiency increased microglia-mediated phagocytosis of synaptic elements and altered oxylipin signaling in the rodent developing brain, thereby altering neuronal morphology and affecting the cognitive performance of the offspring (Madore et al., 2020). However, its long-term effects later in adulthood have not been characterized extensively in AD development. Nonetheless, uses of n-3 FA are safe and well-tolerated during different stages of human life cycles, including fetal and postnatal periods that are critical for neurogenesis and myelination (Olsen et al., 1992; Dunstan et al., 2008; Helland et al., 2003; Garcia-So et al., 2019). We examined the role of neuro-immune responses early in life in influencing AD-associated risks long before the appearance of dementia. Our study aimed to define the potential of injectable n-3 TG during the critical window of brain development in influencing microglial lipid metabolism in response to neuroinflammation that may affect AD progression later in life.

## 2 Methods

### 2.1 Materials

The following reagents were used: Omegaven^®^ (Fresenius Kabi), Trizol (Invitrogen). iScript reverse transcriptase (Thermo Cycles Bio-Rad), SYBR PCR kit (Thermo Fisher Scientific), Phosphate-buffered saline (PBS) (Fisher Cytiva), Heparin (Sigma), 4′,6-diamidino-2-phenylindole (DAPI) (Invitrogen), GFAP antibody (Invitrogen), Ionized calcium-binding adapter molecule 1 (Iba1) antibody (Wako), Triggering receptor expressed on myeloid cells 2 (Trem2) antibody (Invitrogen), Alexa Fluor^™^ 488 (Invitrogen), Alexa Fluor^™^ Plus 647 (Invitrogen), Alexa Fluor^™^ 594 (Invitrogen), Xylene (Fisher Scientific), Citrate Buffer (Sigma-Aldrich), VECTASTAIN Elite ABC Reagent (Vector), DAB Substrate Kit (Vector), Hematoxylin (Abcam), Hydrochloric acid (Fisher Scientific), Paraformaldehyde (PFA) (Fisher Scientific).

### 2.2 Animal models and treatment

The anti-inflammatory effects of n-3 TG in microglial cells were assessed using hemizygous mice (5xFAD) [B6SJL-Tg(APPSwFlLon, PSEN1M146LL286V) 6799Vas/Mmjax, Stock number 34840-JAX, MMRRC] an AD mouse model which expresses human APP and PSEN1 transgenes with five AD-linked mutations (Oakley et al., 2006). These mice recapitulate many aspects of AD pathology, such as amyloid plaques and neuron loss, and display a range of motor and cognitive deficits. 5xFAD hemizygous mice and their corresponding wild-type (WT) littermates were generated by crossing 5xFAD mice with F1 (B6SJLF1/J) mice obtained from the Jackson Laboratory. To examine the impacts of n-3 supplementation during brain development, 5xFAD dams were IP injected with a selected high dose (1.5 g n-3 TG/kg BW) of fish-oil-based lipid emulsion, Omegaven, based on our previous report that was tested in both male and female mice and rats, exhibiting no notable adverse effects (Hu et al., 2021), at gestation (E) day 14, 16, and 18. The gestational days were chosen because E14 marks the beginning of microglial colonization in the brain (Matcovitch-Natan et al., 2016), while E18 coincides with the final stages of neurogenesis and neuronal differentiation in mice, making this window critical for brain development (Stepien et al., 2020). The animals were housed in Columbia University’s Animal Facilities, adhering to a 12-h light/12-h dark cycle at room temperature. All experimental procedures conducted received approval from the Institutional Animal Care and Use Committee at Columbia University.

### 2.3 Quantitative real-time PCR

Total ribonucleic acid (RNA) was isolated from the whole brain using Trizol reagent (Invitrogen). Single-stranded complementary deoxyribonucleic acid (cDNA) was synthesized using iScript reverse transcriptase (Bio-Rad). Relative quantification of messenger ribonucleic acid (mRNA) expression was determined by real-time qPCR with Fast SYBR Green PCR kit (Applied Biosystems) normalized to a housekeeping gene GAPDH on Thermal Cyclers (Bio-Rad). The difference between the target gene and the housekeeping gene Ct value (ΔΔCt) was calculated for all experiments. The expression of the target gene (linear value) was normalized to the housekeeping gene, which was determined by 2^−ΔΔCt^ (Livak and Schmittgen, 2001).

### 2.4 Behavioral and cognitive assessments

Behavioral and cognitive assessments were conducted to assess the potential cognitive improvement among 5xFAD mice that were administered bolus n-3 TG treatments during perinatal brain development at selected ages determined by previous reports (Forner et al., 2021) and our AD model validation results. All these behavioral tests were performed at the Mouse NeuroBehavior Core facility at Columbia University. Wide range of studies confirms that neuronal loss occurs in 5xFAD mice between 6 and 12 months of age (Jawhar et al., 2012; Heraud et al., 2014; Eimer and Vassar, 2013; Fiol-deRoque et al., 2013). More recent studies have revealed that synaptic degeneration and notable neuronal loss can start as early as 4–5 months (Forner et al., 2021; Kim et al., 2019). We performed the Open Field (OF) tests to assess the spontaneous exploratory behavior of n-3-treated mice in a new environment, including locomotion and anxiety-related behaviors (Yang et al., 2012). Each 4-to 5-month-old mouse had a 30-min session monitored by Activity Monitor Version 7 software (Med Associates Inc.). It was then placed in the center of a Plexiglas arena (27.31 × 27.31 × 20.32 cm, Med Associates ENV-510) under dim light (~5 lux) for free exploration. Infrared beams along the arena’s axes tracked movement, stereotypies, and time in the center zone (14.29 × 14.29 cm). Data were analyzed in six 5-min intervals. Arenas were cleaned with 70% ethanol and dried between trials. We performed the Y-Maze to assess short-term spatial memory in 4-month-old mice by leveraging their innate exploration behavior (Sukoff Rizzo et al., 2018). In the Y-maze experiment, mice were put to explore three arms with equal dimensions (35 cm long, 5 cm wide, 10 cm high), each marked with a different sticker (equal sign, bus, plane) (Cho et al., 2021). During Trial 1, mice were familiarized with two arms for 10 min, separated by an opaque door, then placed in a holding cage for 10 min. In Trial 2, mice explored all three arms for 5 min. The movement was recorded using Ethovision XT 12 software. The preference score, indicating memory performance, is calculated as: preference score = [time in novel arm/(time in novel arm + time in the familiar arm)] × 100.

Neonatal mouse pups emit ultrasonic vocalizations (USV) (Colombo et al., 2023). This behavior was tested on postnatal days P4, P6, P8 and P10 aEer perinatal n-3 treatment. Each pup was briefly isolated in a plastic container (10 cm × 8 cm × 8.5 cm) with fresh bedding and immediately placed in a soundproof chamber (Med Associates). After a 3-min recording, pups were marked for identification and returned to their nest. Ultrasonic calls were captured using a sensitive microphone and analyzed with Ultrasound Microphone (Avisoft UltraSoundGate condenser microphone CM16, Avisoft Bioacoustics).

### 2.5 Morphological and immunohistochemistry analysis

Following maternal n-3 TG treatment, whole brains of the young (7-day-old) or adult (4-5-month-old) 5xFAD mice were isolated for histological and biochemical analysis of AD pathology hallmarks. Mouse brains were extracted and fixed in 4% PFA for 24–48 h at RT. Fixed brains were then embedded in paraffin. The thickness of the brain section is 10 μm. The paraffin-embedded section was deparaffinized by incubating with xylene and rehydrated with 100% ethanol followed by another incubation with 95% ethanol and 70% ethanol. The antigen was retrieved in a heated 10 mM Citrate buffer (Invitrogen). The sections were rinsed in PBS for 10 min, followed by blocking with serum for 60 min at RT. Samples were incubated overnight with primary antibodies with a concentration of 1:500 for Anti-Beta-Amyloid 1–42 antibody (AB5078P, Sigma), and Anti-Amyloid Antibody, *β* 1–40 (AB5074P, Sigma), and subsequently incubated with secondary antibodies (biotinylated goat anti-rabbit IgG) for 60 min at RT. Sections were then incubated with VECTASTAIN Elite ABC Reagent at RT for 30 min, followed by incubation with the substrate working solution at RT for 0.5–1.0 min. As previously described, sections were scanned and imaged using a Leica SCN400 whole-slide digital imaging system (Chang et al., 2018). The image analysis was performed using QuPath open-source software platform (Bankhead et al., 2017). Scanned images were set as ImageType “BRIGHTFIELD_H_DAB.” Three to five ROI regions in the cortex and hippocampus were highlighted, and the QuPath’s positive cell count function was used to quantify cells with positive Aβ staining. The results are expressed in the percentage of positive signals out of the total cells detected by hematoxylin (%).

In a separate study, the sections were incubated overnight at 4°C in with the primary antibodies for probing Iba1 (1:150), GFAP (1:150), and Trem2 (1:150) and corresponding secondary antibodies (Alexa Fluor^™^ 488, concentration 1:200; Alexa Fluor^™^ 594, concentration 1:200; Alexa Fluor^™^ 647, concentration 1:200) and DAPI (concentration 1:1000) for 1 h at RT. The stained slides were imaged using a Nikon AXR Confocal microscope. The DAPI, Iba1, GFAP, and Trem2 positive cell regions in the cortex and hippocampus were analyzed by ImageJ (Fiji) software (version 2.14.0/1.54f) available online from the National Institutes of Health (NIH). Selected areas were visualized through the “threshold” and quantified with the “Analyze Particle” functions as previously outlined (Chang et al., 2009; Chang et al., 2014).

### 2.6 Lipidomic

The employed lipidomic investigation and analytical workflow utilized a state-of-the-art LC–MS/MS platform, integrating an Agilent 6,490 Triple Quadrupole (QQQ) MS with an Agilent 1,260 Infinity LC system at the Columbia Biomarker Core Laboratory (Area-Gomez et al., 2021). Briefly, lipid extracts were prepared from cell lysates using a chloroform-methanol extraction method and spiked with appropriate internal standards and analyzed on a platform comprising Agilent 1,260 Infinity HPLC integrated to Agilent 6490A QQQ mass spectrometer controlled by Masshunter v 7.0 (Agilent Technologies) as described before (Chan et al., 2012). Tree panels, scanning for either positive lipids, negative lipids or neutral lipids (under positive mode), were analyzed. Equal amounts of internal standards with known concentrations were spiked into each extract. Each standard was later used to calculate the concentrations of corresponding lipid classes by first calculating the ratio between the measured intensities of a lipid species and that of the corresponding internal standard multiplied by the known concentration of the internal standard. Lipid levels for each sample were calculated as nmol normalized for protein concentration (nmol/mg protein). The abbreviations of the names of the different classes of lipids are summarized in Supplementary Table 1.

### 2.7 Statistical analyses

All data are expressed as mean ± SEM. Statistical differences between group means were assessed by Student’s *t*-tests to compare endpoints or ANOVA with post-hoc tests to evaluate potential interactions between groups using GraphPad Prism 9.5.0. The variances are considered equal determined by F-tests. All statistical tests assumed a 95% level of confidence of a normal distribution (Shapiro–Wilk normality tests). For all tests, differences were considered significant when *p* < 0.05 and four significance levels are indicated as follows: *p < 0.05, **p < 0.01, ***p < 0.001, ****p < 0.0001.

## 3 Results

### 3.1 Early n-3 treatment effects on neuroinflammation

**Figure 1:**
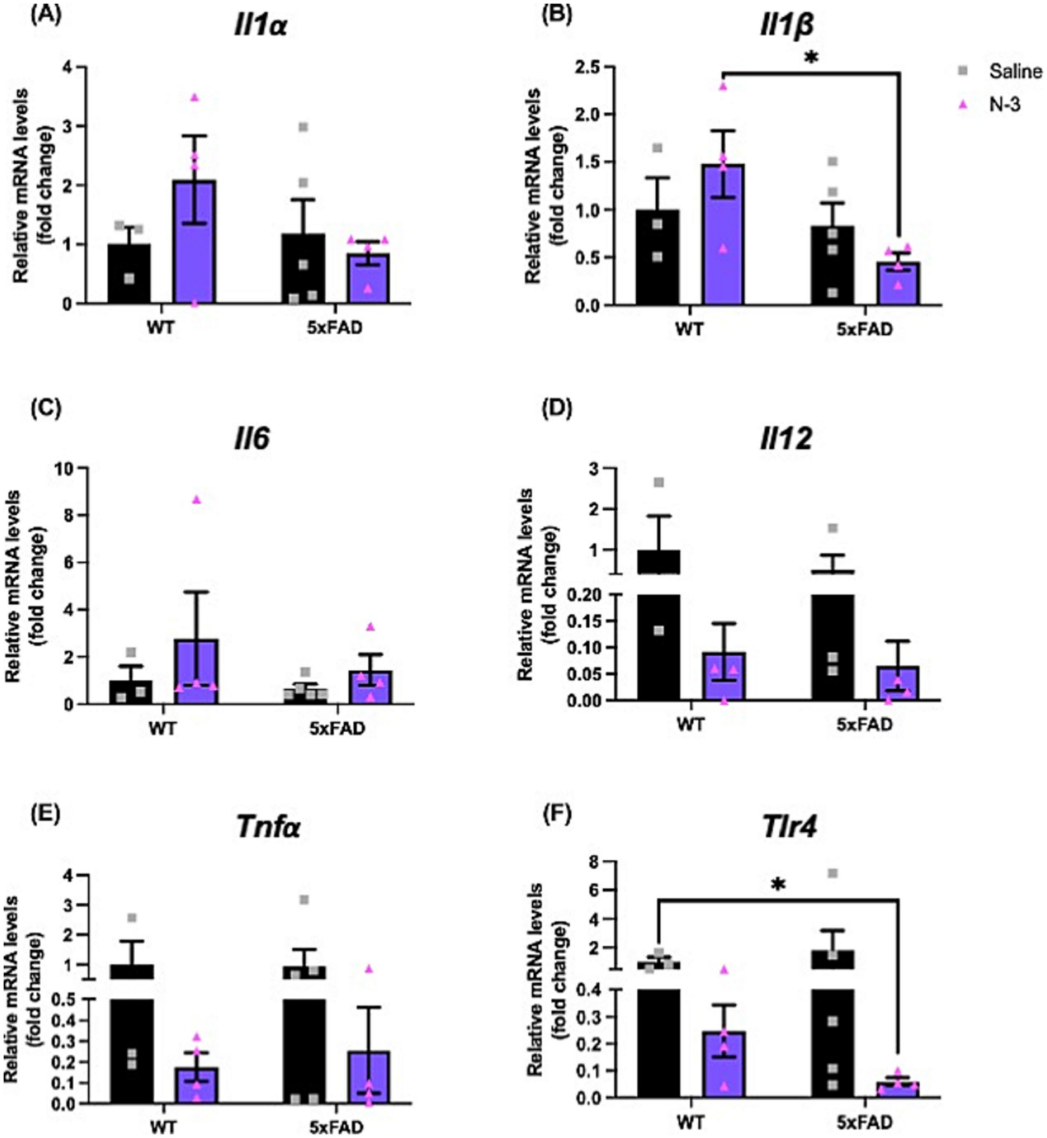
Early n-3 treatments affect pro-inflammatory cytokine expression in young 5xFAD mice. WT and 5xFAD dam received saline or n-3 injections at E14, E16, and E18. Transcriptional expression of (A) *Il1α*, (B) *Il1β*, (C) *Il6*, (D) *Il12*, (E) *Tnfα*, and (F) *Tlr4* in 7-day-old offsprings were determined by real-time PCR (qPCR) as described in the “Methods” (n: Saline WT = 3, Saline 5xFAD = 5, n-3 WT = 4, n-3 5xFAD = 4). Two-way ANOVA, Sidak’s multiple comparisons test. Mean ± SEM. *p < 0.05.

As initially described by Oakley et al. (2006) 5xFAD mice exhibited aggressive AD pathological hallmarks, including amyloid plaques, gliosis, and neuron loss in multiple brain regions as early as at 2 months of age, and displayed a range of cognitive and motor deficits (Eimer and Vassar, 2013). Importantly, 5xFAD recapitulates many neuroinflammation-related phenotypes (Oakley et al., 2006) such as astrogliosis and microgliosis (Rodriguez-Urgelles et al., 2022) in AD development. To examine the impacts of n-3 TG during early brain development in AD, hemizygous 5xFAD dams were injected with n-3 TGRP at gestation days E14, E16, and E18. We compared the pro-inflammatory levels following treatment with either saline or n-3 intervention in these 7-day-old 5xFAD mice. In WT mice, there was no significant difference in the expression of pro-inflammatory cytokines at an early age (7 days, ns). Perinatal administration of n-3 TG resulted in a notable trend in decreased expression of major pro-inflammatory cytokines such as interleukin-1 alpha (*Il1α*) and *Il1β* (ns, Figures 1A,B) in 5xFAD mice, n-3 treatment also reduced overall expression of *Il12, Tnfa*, or toll-like receptor 4 (*Tlr4*), regardless of the genotype (Figures 1D–F, ns).

**Figure 2:**
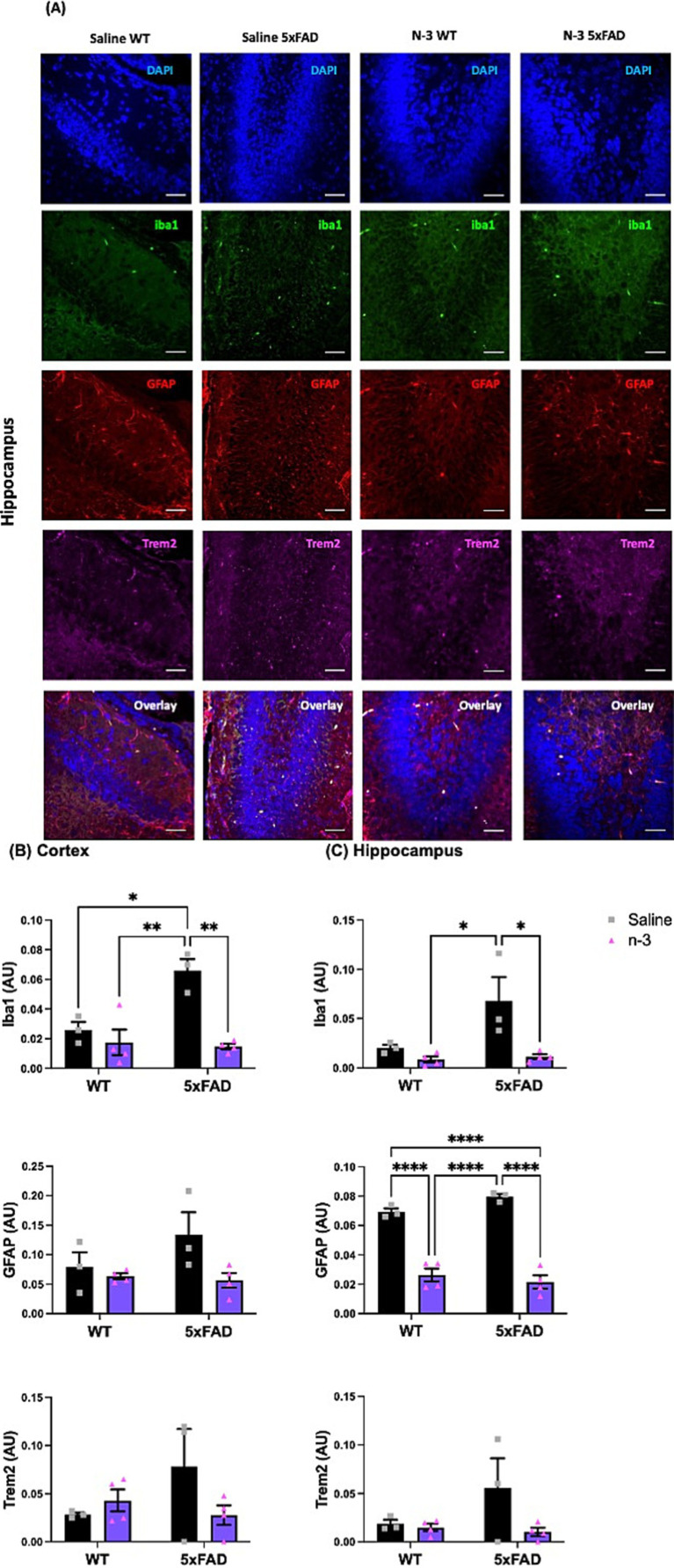
Early n-3 treatments reduce gliosis-associated marker levels in the young 5xFAD offspring. WT and 5xFAD dams received saline or n-3 injections at E14, E16, and E18. Brains of 7-day-old offspring were isolated, fixed, and stained for DAPI, Iba1, GFAP, and Trem2 by IFC. **(A)** Representative images (scale bar = 0.5 μm) and quantitation of Iba1-, Trem2-, and GFAP-expressed in the **(B)** cerebral cortex and **(C)** hippocampus in the same microscopic fields (*n* = 3–6). Two-way ANOVA, Sidak’s multiple comparisons test. Mean ± SEM. *p < 0.05, **p < 0.01, ****p < 0.0001.

To further characterize the impacts of perinatal n-3 TG treatment on neuroinflammation in the developing brain, markers for microgliosis and astrogliosis in the cortical layer (Supplementary Figure 1) and hippocampus (Figure 2) were examined by immunofluorescence (IFC). Our data show that the non-treated (NT) 5xFAD mice had increased levels of Iba1, GFAP, and Trem2 in the hippocampus and cortex regions when compared to the NT WT littermates. Perinatal n-3 injections suppressed gliosis-associated expression of Iba1 in the cortex (Figure 2B) and hippocampus (Figure 2C) compared to saline-injected control mice. A significant reduction in GFAP levels was observed in the hippocampus region of WT and 5xFAD mice (Figure 2C). A similar trend in decreased Trem2 levels was observed in n-3 treated 5xFAD groups.

**Figure 3:**
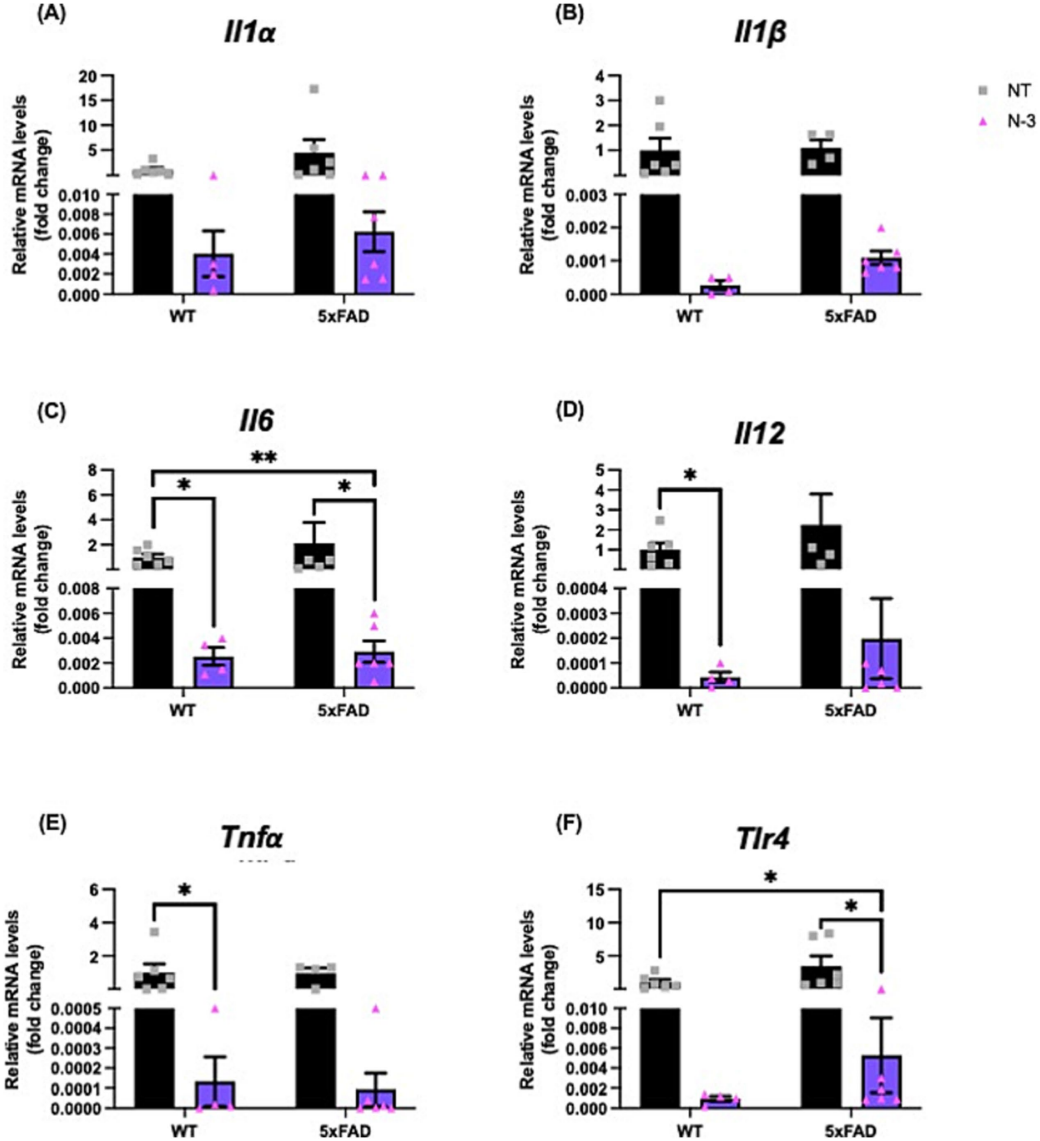
N-3 treatments decrease mRNA expression of pro-inflammatory markers in adult 5xFAD mice. Transcriptional expression of **(A)** *Il1α*, **(B)** *Il1β*, **(C)** *Il6*, **(D)** *Il12*, **(E)** *Tnfα*, and **(F)** *Tlr4* in 4-5-month-old WT littermates and 5xFAD mice underwent maternal treatment of saline or n-3 treatment determined by qPCR as described in the “Methods” (n: NT WT = 6, NT 5xFAD = 7, n-3 WT = 4, n-3 5xFAD = 6). Two-way ANOVA, Sidak’s multiple comparisons test. Mean ± SEM. *p < 0.05, **p < 0.01.

**Figure 4:**
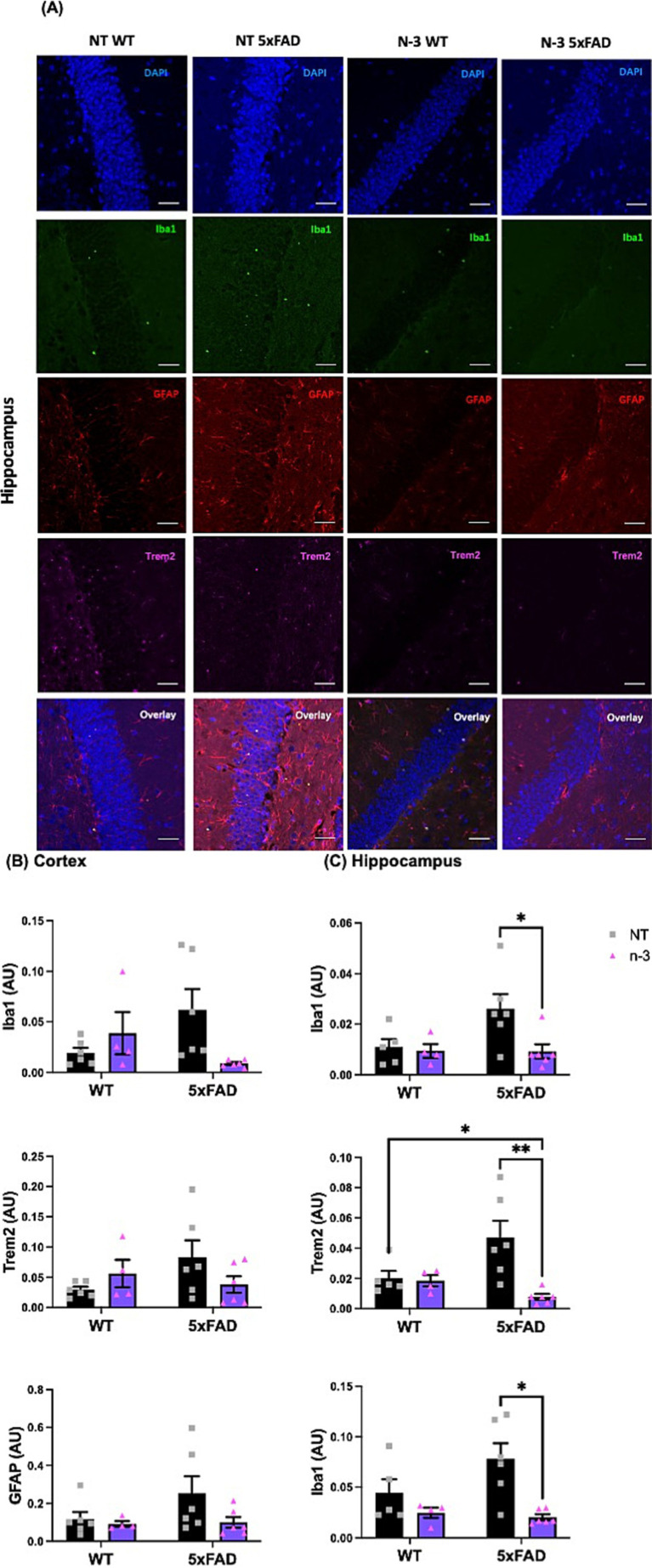
Early n-3 treatments decrease the expression of gliosis-associated markers in adult 5xFAD mice. WT and 5xFAD dam received saline or n-3 injections at E14, E16, and E18. Brains of 4-5-month-old offspring underwent maternal treatment of saline or n-3 treatment determined were isolated, fixed and stained for DAPI, Iba1, GFAP, and Trem2 by IFC. **(A)** Representative images (scale bar = 0.5 μm) and quantitation of Iba1-, Trem2-, and GFAP-expressed cells in the **(B)** cerebral cortex and **(C)** hippocampus in the same microscopic fields (*n* = 3–6). Two-way ANOVA, Sidak’s multiple comparisons test. Mean ± SEM. *p < 0.05, **p < 0.01.

We continued to monitor the development of the neuroinflammatory phenotypes of these AD 5xFAD through adulthood. At the age of 4–5 months, 5xFAD mice exhibited similar expression levels of pro-inflammatory markers, including *Il1β*, *Il6*, interleukin-12 (*Il12*), and *Tlr4* (Figure 3) when compared with their WT littermates. Perinatal administration of n-3 TG markedly decreased pro-inflammatory cytokine mRNA expression (Figures 3A–F) in both WT and 5xFAD mice. Expression of *Il6*, *Il12* and *Tnfα* were significantly reduced in n-3 treated WT (Figures 3C–E). Expression of *Il6* and *Tlr4* was significantly reduced in adult 5xFAD mice compared to that in the NT 5xFAD mice (Figures 3C,F). Our findings on the gliosis development (Figure 4 and Supplementary Figure 2) show that NT 5xFAD had higher expression levels (ns) of gliosis markers than WT littermates, including Iba1, Trem2 and GFAP in both cortex and hippocampus (Figures 4B,C). Perinatal n-3 treatment significantly reduced the levels of Iba1, Trem2, and GFAP in the hippocampus of treated 5xFAD mice compared to NT 5xFAD mice (Figure 4C). Our findings suggest that bolus n-3 treatment at the perinatal period may offer sustainable effects on influencing brain cellular immune responses.

### 3.2 N-3 TG effects on cognitive deficits in AD mice

**Figure 5:**
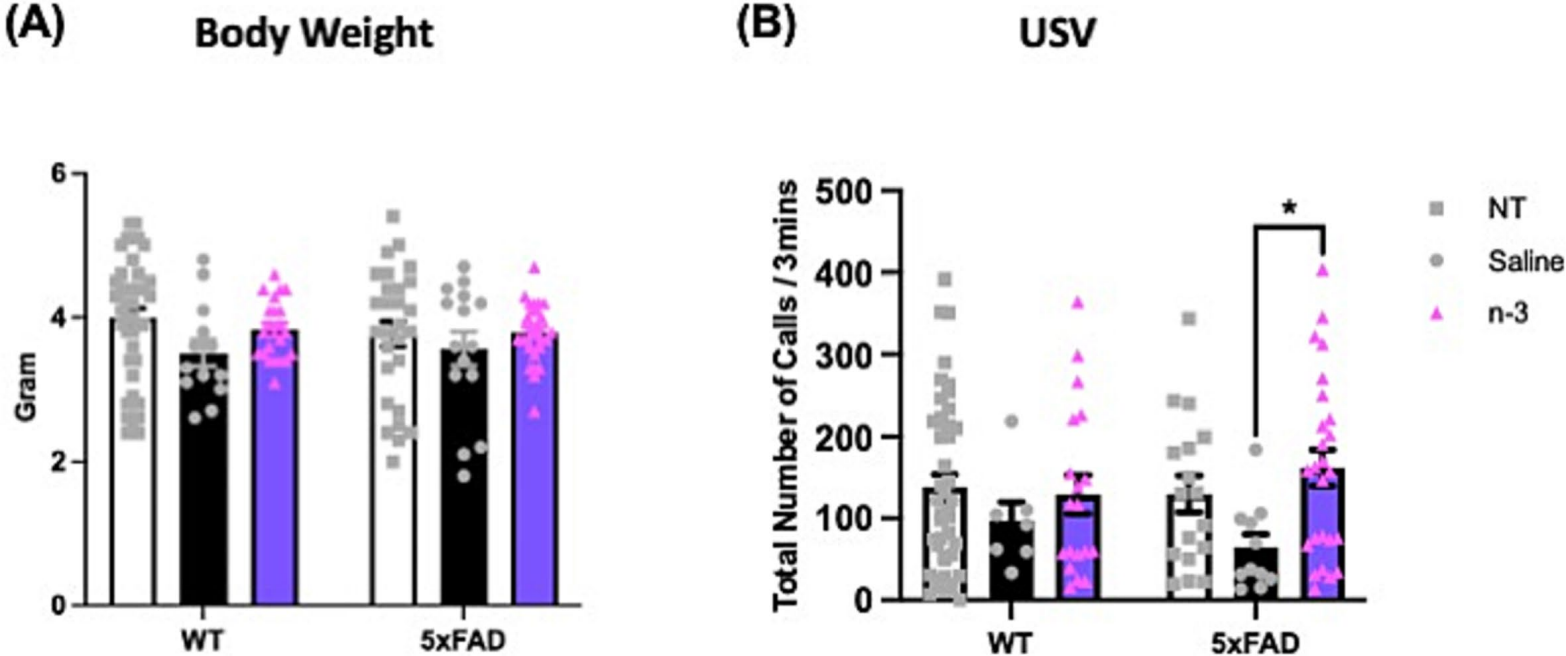
Assessment of ultrasonic vocalizations (USV) in 6-day-old WT and 5xFAD mice. **(A)** Body weight of WT and 5xFAD pups at postpartum (P) 6. **(B)** Total USV calls per 3 min in NT, prenatal saline-, and n-3-treated offspring at P6 (n: NT WT = 23, NT 5xFAD = 17, Saline WT = 7, Saline 5xFAD = 11, n-3 WT = 19, n-3 5xFAD = 25). Two-way ANOVA, Sidak’s multiple comparisons test. Mean ± SEM. *p < 0.05.

To further characterize the impacts of perinatal n-3 TG treatment on the developing brain, high-frequency USV emitted by young offspring was recorded to evaluate social communication, distress, or emotional states (Premoli et al., 2021). We first validated the method by assessing the USV of mouse pups at early developmental stages, specifically at 4, 6, 8, and 10 days post-natal. Our analysis revealed that both groups exhibited a peak in the number of USV at 6 days old (Supplementary Figure 3). Consequently, we evaluated the USV of the pups at postnatal day 6 in mice with maternal n-3 treatment. With a similar body weight (Figure 5A), our data indicate that both male and female NT pups had fewer call numbers than treated 5xFAD pups. There is a significant increase in call numbers in n-3 treated pups compared to saline-treated pups (Figure 5B). Body weight data in Supplementary Figure 4 show that male saline-treated WT pups had lower body weight compared to NT WT pups, while no significant differences were observed in female pups or 5xFAD pups.

**Figure 6:**
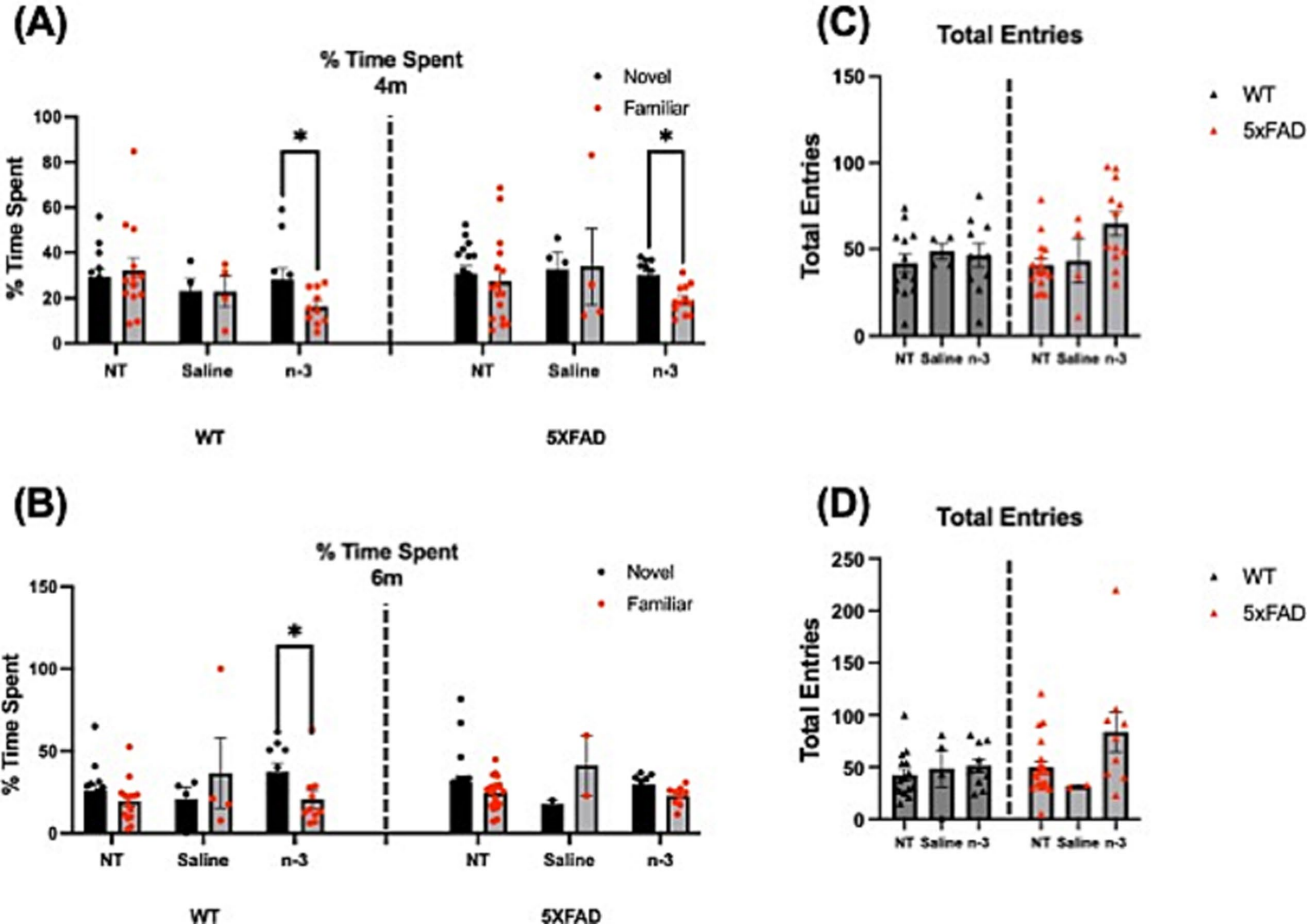
N-3 effects on mouse cognitive and spatial memory capacities. WT and 5xFAD mice at 4 and 6 months of age were tested using the Y-maze test. A comparison of the percentage of time spent between the novel and familiar arms in **(A)** 4-month and **(B)** 6-month NT, saline-and n-3-treated WT and 5xFAD mice was recorded. The total frequency of entrances to the novel arm in **(C)** 4-month and **(D)** 6-month WT and 5xFAD mice with perinatal saline or n-3 treatment (n: NT WT = 13, NT 5xFAD = 16, Saline WT = 4, Saline 5xFAD = 4, n-3 WT = 10, n-3 5xFAD = 12). Two-way ANOVA and Sidak’s multiple comparisons test. Mean ± SEM. *p < 0.05.

We monitored these mice as they matured into adulthood and evaluated AD-associated cognitive and behavioral impairment by the Y-maze and OF maze tests on 5xFAD mice from the ages of 3 months. We first validated the methods for Y-maze spontaneous alternation in F1 mice to evaluate changes in spatial working memory and exploratory behavior. The results on percent alternation and total arms entries reached the expected values at 60% and ~60 entries, respectively (Supplementary Figures 5A,B). A lack of significant difference in the percentage of time spent between novel and familiar arms (Figures 6A,B) and total entries (Figures 6C,D) was observed in the NT and saline-treated WT and 5xFAD groups across different ages from 4 to 10 months. A significant increase in time spent exploring the novel arm was observed in WT and 5xFAD mice receiving perinatal n-3 treatment, suggesting improved spatial memory and exploration behavior at 4 and 6 months (Figures 6A,B). At 10 months of age, with no significant changes in total entries, an increase in time spent exploring the novel arm was observed in WT mice receiving perinatal n-3 treatment (Supplementary Figure 6).

**Figure 7:**
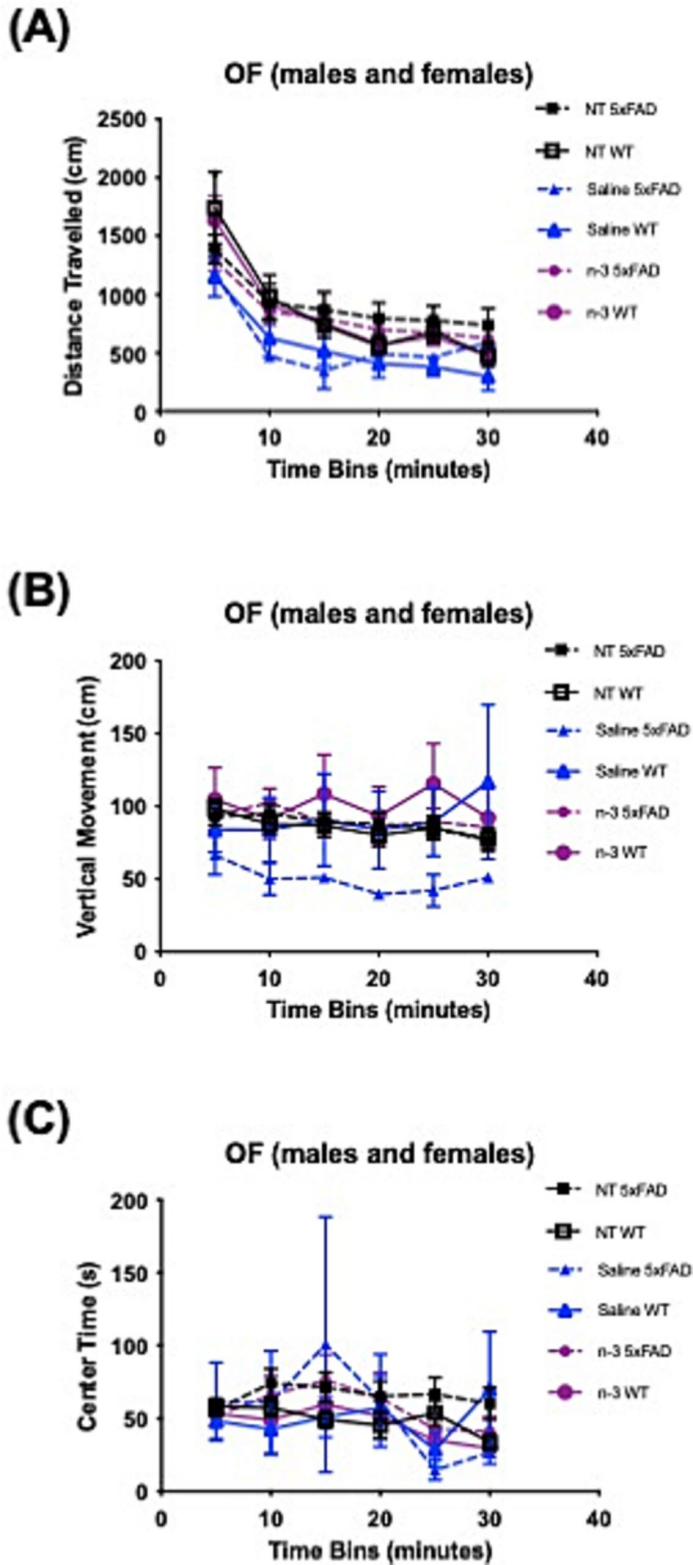
Assessment of locomotor activity and exploratory behavior in WT and 5xFAD mice. **(A)** Distance traveled across 40-min time bins in the OF test was recorded for NT, saline-and n-3-treated 5xFAD and WT mice. **(B)** Vertical movement across 40-min time bins in the OF in NT, saline-and n-3-treated 5xFAD and WT mice at 5–6 months old. **(C)** The time spent in the center of the OF across 40-min time bins in the OF was recorded for NT, saline-and n-3-treated 5xFAD and WT mice (n: NT WT = 24, NT 5xFAD = 36, Saline WT = 4, Saline 5xFAD = 2, n-3 WT = 8, n-3 5xFAD = 12). Unpaired *t*-test. Mean ± SEM.

We have also performed OF maze test to assess locomotor activity, exploratory behavior, and anxiety of adult 5xFAD mice. Each mouse ran one trial for 30 min. The mouse was placed in the center of the arena and roamed freely in the chamber. Total distance traveled (Figure 7A), vertical movement (Figure 7B), and center time (Figure 7C), were recorded. No notable changes were observed for mouse locomotor activity or exploratory behavior regardless of the genotype, at 5–6 months of age, or sex (Supplementary Figure 7). NT 5xFAD mice displayed a significantly higher travel distance and increased center time than NT WT only at 10 months (Supplementary Figures 8A,C).

**Figure 8:**
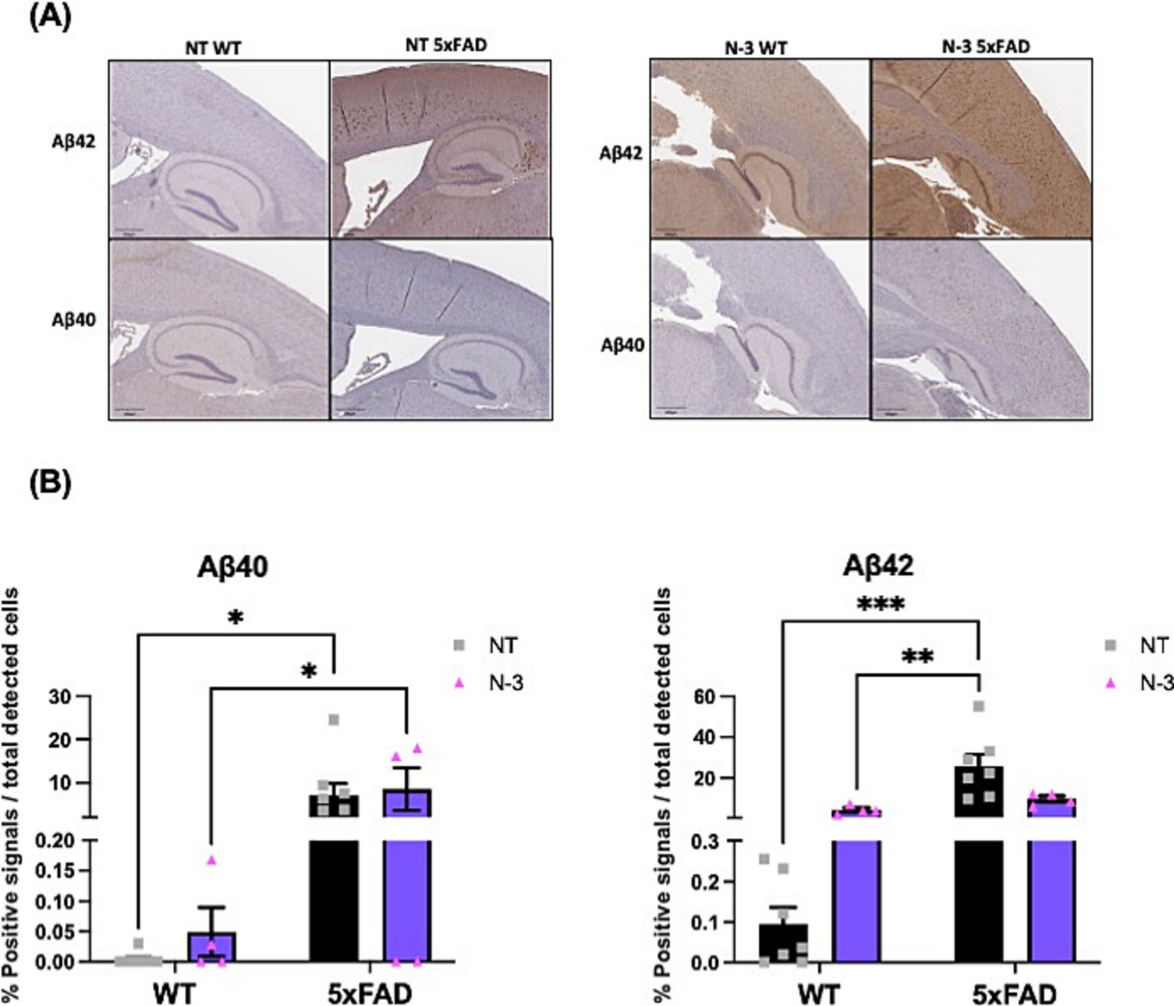
Deposition of amyloid plaques in 5-month-old 5xFAD mice. **(A)** Representative images and **(B)** quantitation of Aβ40 and Aβ42 plaque formation from 5-month-old WT and 5xFAD mouse with or without n-3 treatment by immunohistochemical analysis. The brown-red dots represent plaque deposition in Aβ. The results are expressed in the percentage of positive signals out of the total cells detected by hematoxylin (%) as described in the “Methods” section (*n* = 4–8). Two-way ANOVA, Sidak’s multiple comparisons test. Mean ± SEM. *p < 0.05, **p < 0.01, ***p < 0.001.

Although that lack of consistent cognitive deficits has been observed in these 5xFAD mice, pathological amyloid deposition has been detected in 5xFAD mice at the age of 5 months when compared to the WT littermates, indicated by the notable Aβ plaque deposition detected by Aβ 40 and Aβ 42 (Figures 8A,B). Quantification on Aβ 40 and Aβ 42 confirms no significant distinctions were observed between the NT and n-3-treated mice.

### 3.3 N-3 TG rapidly and sustainably alter CNS lipid composition

**Figure 9:**
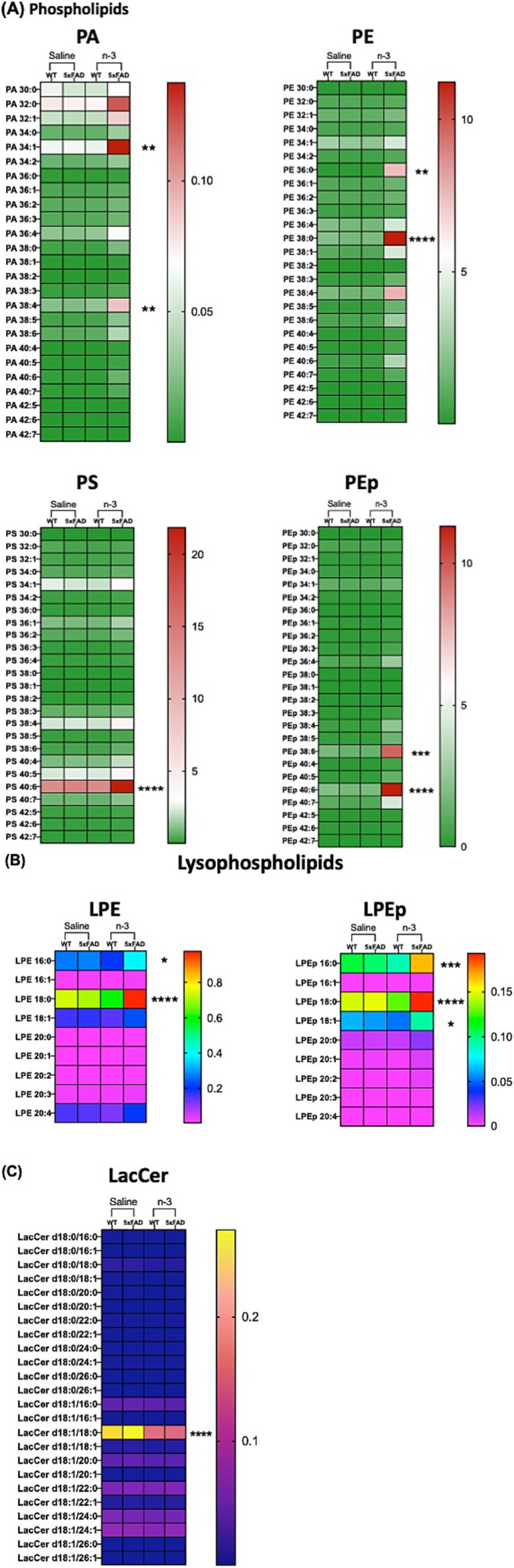
N-3 treatments modulate CNS lipid composition in 5xFAD mice. Lipidomic analysis was conducted on brain samples from WT and 5xFAD mice at P7 following perinatal saline or n-3 FA treatment. Lipid profiles were determined as described in the “Methods” section **(A)** PL, **(B)** lysoPL, and **(C)** LacCer. *n* = 3–4. Two-way ANOVA, Sidak’s multiple comparisons test between WT and 5xFAD mice treated with saline or n-3. Mean ± SEM. *p < 0.05, **p < 0.01, ***p < 0.001, ****p < 0.0001.

To determine whether changes in FA and specific membrane lipid species are responsible for initiating inflammatory signaling pathways, 32 major lipid species in whole brain lysates, including TG, phospholipid (PL), free cholesterol (FC), cholesteryl esters (CE), and sphingolipids, were extracted and analyzed using LC–MS/MS platform (Supplementary Table 1 and Supplementary Figure 9). Our data demonstrate compositional changes in cellular lipid species in 5xFAD-P7 offspring who received perinatal injectable n-3 treatment when compared to NT and saline-treated WT and 5xFAD mice. Major membrane lipid species, i.e., PL (PA, PS, PE, PEp) (Figure 9A) and lysophospholipids (lysoPL) (LPE, LPEp) (Figure 9B) were increased after n-3 treatment. Analysis of sphingolipids reveals that the total lactosylceramide (LacCer) content of mice treated with n-3 was significantly lower than that in mice treated with saline (Figure 9C). LacCer, a complex membrane-associated proinflammatory glycosphingolipid (Yu et al., 2021), has been shown to induce neurodegeneration by activating astrocytes and neuroinflammation that perturb neuron survival. We speculate that the potential beneficial effects of n-3 TG in ameliorating brain inflammatory challenges could result from suppressing the production of pro-inflammatory lipid species while simultaneously increasing anti-inflammatory and pro-resolution lipid mediators.

## 4 Discussion

Our studies aimed to determine the effects of n-3 FA treatment in AD mice on modulating brain inflammatory markers, cognitive function, and lipid composition during development and later in adulthood. Our results indicate that bolus injections of n-3 during gestation in mice reduced several major pro-inflammatory cytokines in the offspring. In addition, n-3 treatment significantly suppressed gliosis markers, such as Iba1 and GFAP, in the cortical and hippocampal regions of the mouse brain. n-3-treated mice demonstrated partial improvements in spatial memory and behavioral activity in cognitive tests compared to the untreated group. N-3 treatment also altered the lipid composition of the brain, reducing the levels of pro-inflammatory lipids such as LacCer, which may have a protective effect on the brain by inhibiting inflammatory signaling pathways. Notably, perinatal n-3 treatment also exhibits effects on modulating levels of several inflammatory genes, gliosis markers, and spatial memory in WT adult offspring. This indicates that n-3 FA may serve as a potential therapeutic agent by modulating both neuroinflammation and lipid composition, which are critical factors in the brain development and progression of AD pathology.

Transgenic 5xFAD mice have been widely utilized in the investigation of AD-related pathologies. Although the original report indicates that a pro-inflammatory response can be detected as early as 2–3 months in 5xFAD mice (Oakley et al., 2006; Manji et al., 2019), our research revealed that the disparities between the WT and 5xFAD groups were observed only at the age of 4–5 months with the increased expression of pro-inflammatory markers in these transgenic mice. We were able to observe the dysregulation of pro-inflammatory markers and the progression of Aβ plaque accumulation at a similar age of AD mice, which is consistent with several previous reports (Oakley et al., 2006).

In the present study, n-3 treatment showed a trend of reducing pro-inflammatory cytokine expression in 7-day-old 5xFAD offspring, although statistical significance was not observed for some markers. Previous studies have reported the beneficial effects of n-3 FA in reducing inflammation and promoting neuroprotection by feeding or oral supplements in adult mice (Lim et al., 2005; Green et al., 2007). However, no study has shown the effects on influencing expression of pro-inflammatory cytokines in 7-day-old 5xFAD mice after maternal n-3 treatment by IP injections. It has been postulated that the administration of n-3 DHA emulsion via injection initially targets the liver for uptake. Subsequently, the liver metabolizes the emulsion, resulting in its secretion into the plasma where it contributes to pools of lysophosphatidylcholine and non-esterified FA (Nguyen et al., 2014). This process ultimately facilitates the transport of DHA to the brain (Nguyen et al., 2014). Future studies will include the optimal dose and timing of injections in adult animal models to pioneer innovative delivery systems for bioactive lipids to ameliorate neuroinflammation related to CNS disorders. Notably, previous studies have investigated the long-term impact of prenatal n-3 supplementation in a Parkinson’s disease model, where it was shown to influence neuroinflammation and dopaminergic neuronal integrity in offspring (Delattre et al., 2017). This suggests that the effects of prenatal n-3 supplementation may not only depend on the disease context but also involve shared lipid metabolic and neuroinflammatory mechanisms. Future comparative studies across neurodegenerative disease models could provide valuable insights into the systemic effects of n-3 FA on brain health. Nonetheless, as we emphasize the importance of bolus n-3 treatment in preventing CNS disorders, a combined regimen of bolus n-3 injections followed by oral supplements may offer complementary mechanisms for brain inflammatory and metabolic homeostasis, providing synergistic protection after inflammatory insult.

Our findings suggest that n-3-associated beneficial effects in reducing neuroinflammation may be mediated by lowering GFAP levels in the hippocampus. We found the reduction in GFAP levels following n-3 treatment indicates its potential effectiveness in reducing neuroinflammation and maintaining nervous system health. GFAP, a key marker of astrocyte activation in the inflammatory state that causes neuronal damage (Han et al., 2019). Consistently, previous studies have demonstrated that the number of GFAP-positive cells in the hippocampus of 5xFAD mice was significantly decreased by oral administration of n-3 (Park et al., 2020). These studies further support the potential therapeutic effect of n-3 in addressing neuroinflammation by reducing GFAP expression in the hippocampus. In addition, although we have not observed sex differences in our AD model validation studies (Supplementary Figures 3, 4, 7), it has been reported that female mice are prone to a higher number of plaques than male mice in Aβ accumulation (Forner et al., 2021). 5xFAD mice may display sex-dependent inflammatory responses at a later age; it is an ongoing interest to explore the sex difference that may interfere with immune cell phenotypes and response in CNS.

Previous studies have demonstrated that n-3 FA have a positive effect on the improvements of spatial learning and memory performance (Su, 2010). It seems probable that during the development of mice, the synthesis and absorption of DHA by cell membrane PL could facilitate the growth and formation of synapses and the development of nerve cells (Su, 2010). Our results from the Y-maze tests demonstrated improved spatial memory and learning abilities following perinatal n-3 treatment in 5xFAD mice, as indicated by increased exploration of the novel arm. Additionally, n-3 treatment increased USV levels in mouse pups at postpartum day 6, potentially associated with enhanced social interactions, an effect not previously reported for 5xFAD mice. However, our Y-maze and OF assessments did not differentiate the phenotypes between the AD mutants and WT littermates. Nonetheless, the results on the Y-maze could be confounded by issues related to motivation and spontaneous activity. It was reported that 5xFAD mice develop distinct phenotypes at different ages. For example, early studies have shown that 5xFAD mice exhibit amyloid deposition as early as 2–4 months (Oakley et al., 2006; Eimer and Vassar, 2013), which is commonly associated with impairments in cognitive function and locomotion. However, other research suggests that certain behavioral deficits, such as learning and memory impairments, become more evident only at later ages, such as >12 months (O’Leary and Brown, 2022). While impaired learning performance can be observed in 5xFAD mice as early as 6–9 months (Yang et al., 2018), these deficits are further confounded by motor impairments at 12–15 months (O’Leary and Brown, 2022; O’Leary et al., 2018). Spatial learning and memory deficits have also been documented in 9–10-month-old 5xFAD mice using tasks like the active place avoidance test (Merlini et al., 2019). These findings suggest that 5xFAD mice display early pathological features such as amyloid deposition but experience progressive and age-dependent behavioral impairments. Our data (Supplementary Figure 8) from 10-month-old 5xFAD NT mice who exhibited a pattern for longer travel distance in the OF test may also be associated with the phenotype of hyperactivity in the 5xFAD mouse model that has been reported earlier (Oblak et al., 2021). The inconsistency between different reports represents a significant obstacle in defining mechanistic insights into AD pathogenesis for therapeutic development. Furthermore, the age-dependent nature of the observed pathologies complicates results in modeling early-stage AD and developing therapeutic interventions targeting initial pathology.

Our results demonstrate the necessity of considering lipid metabolism and inflammation together with the amyloid hypothesis when studying the pathology of AD. The conventional “Aβ hypothesis,” which posits Aβ accumulation as the principal driving factor behind AD pathology. Expanding the amyloid hypothesis with factors related to lipid metabolism and inflammation is crucial for understanding AD pathogenesis. Our data show that 5xFAD mice exhibited Aβ accumulation as early as the age of 4 months. No significant differences in Aβ staining have been found between the NT and n-3 treated 5-month-old 5xFAD mice. However, maternal treatment of n-3 effectively influenced pro-inflammatory marker levels and improved some cognitive deficits in AD mice. It is conceivable that more prolonged treatment regimens or alternative approaches may be required to unveil substantial disparities in Aβ levels. While current therapeutic strategies, such as anti-amyloid therapies, have shown promise, their limitations underscore the need for complementary approaches that address other pathological mechanisms, such as neuroinflammation, oxidative stress, and synaptic dysfunction. In fact, in other studies with a 3-week short-term fish oil intake, research has demonstrated notable enhancements in PL composition but with no effects on plaque accumulation within the 5xFAD mouse brain (Milanovic et al., 2018). It has been shown that increased Aβ in the brain of AD patients is accompanied by a decrease in phosphatidylethanolamine (PE) (Han et al., 2001). PE and phosphatidylinositol (PI) influence Aβ aggregation and processing by affecting cell membrane properties and signaling pathways. PE enhances Aβ toxicity and impacts its clearance, while PL regulates Aβ uptake and degradation (Nesic et al., 2012). The interplay between these two PL may play a crucial role in the pathogenesis of AD. Alterations observed in specific PL species within the 5xFAD mice following n-3 intake, including PA, PS, PE and PEp, may have an impact on the membrane fluidity and protein-lipid interactions of these cells, both of which are crucial for the maintenance of cellular function and integrity (Lee, 2003). Lastly, our investigation of lysoPL revealed significant differences in LPE and LPEp between n-3 treated 5xFAD mice and their saline-treated counterparts. These modifications have the potential to influence cellular membrane properties and lipid-protein interactions (Guan et al., 1999). Additionally, our results indicate that changes in FA and specific membrane lipid species may be responsible for initiating inflammatory signaling pathways in the CNS. We have found that total LacCer content in mice treated with n-3 was significantly lower than that of mice treated with saline. LacCer is a type of glycosphingolipid, which is a complex molecule found in cell membranes. The increased level of LacCer has been associated with the induction of inflammation in mice (Chatterjee et al., 2021; Yu et al., 2021). Previous studies have shown that LacCer could induce neurodegeneration by activating astrocytes that leads to neuroinflammation and affect neuron survival (Colombo and Farina, 2016). Additionally, increased *Tnfα* could stimulate the release of LacCer and the expression of GFAP in astrocytes (Pannu et al., 2005). The expression of *Tnfα* and levels of GFAP were found to be reduced in n-3 treated 5xFAD mice in comparison to saline-control mice. It is important to note that these alterations represent enduring effects resulting from three IP injections. This underscores the necessity for a comprehensive investigation into the mechanistic connections between lipid metabolism, cell signaling pathways and AD pathogenesis.

The 5xFAD mouse model for AD was used to examine the effects of n-3 fatty acids on inflammation and biomarkers. Although some AD phenotypes were not present in our studies, the administration of n-3 treatment influenced the expression of pro-inflammatory cytokines and gliosis markers, suggesting a potential anti-inflammatory role. Early bolus n-3 treatments were also linked to cognitive improvements in adult mice, even without affecting Aβ accumulation, which challenges the conventional Aβ hypothesis. Lipid analysis revealed complex changes in lipid classes that might have affected the role of n-3 fatty acids on cellular function. Importantly, although clinical trials have shown mixed results, they suggest that n-3 supplementation may be most beneficial in early-stage AD or among individuals with specific genetic profiles, such as APOE4 non-carriers (Yassine et al., 2017). These findings highlight the significance of personalized therapeutic strategies and the necessity for further exploration into biomarkers that can predict responsiveness to n-3 interventions. By integrating our findings with these insights, we propose that combining n-3 treatment with current AD therapies could produce synergistic benefits that enhance the effectiveness of amyloid-related therapies by addressing concurrent neuroinflammatory processes. Furthermore, our results regarding early CNS inflammation response and lipid composition could guide the development of n-3-based interventions tailored to particular AD subtypes or stages (Cunnane et al., 2013). In conclusion, n-3 FA demonstrated the possibility of reducing inflammation, modulating behavior, and influencing lipid profiles in the context of AD, providing valuable insight into potential therapeutic pathways. Future studies investigating these mechanisms could provide a deeper understanding of how prenatal n-3 FA administration affects the long-term trajectory of AD pathology and guide the creation of innovative therapeutic strategies that target lipid and inflammatory pathways.

## Data Availability

The data presented in this study has been deposited in the Dryad repository, DOI: 10.5061/dryad.sn02v6xg7.
