# Supplementary material for: Strategic delivery of omega-3 fatty acids for modulating inflammatory neurodegenerative diseases
Source: Front Aging Neurosci. 2025 Mar 17;17:1535094. doi: 10.3389/fnagi.2025.1535094 (PMC11955621; doi:10.3389/fnagi.2025.1535094)

Supplemental Table 1: CNS lipidomic analysis. The comprehensive list of lipid classes, analyzed through lipidomics and the abbreviations and number of species examined within each class, is indicated in parentheses.

FC	Free cholesterol	PA	Phosphatidic acid (25 species)
CE	Cholesterol ester (20 species)	PC	Phosphatidylcholine (25 species)
AC	Acyl carnitine (9 species)	PCe	Ether phosphatidylcholine (25 species)
MG	Monoacylglycerol (18 species)	PE	Phosphatidylethanolamine (25 species)
DG	Diacylglycerol (28 species)	PEp	Plasmalogen phosphatidylethanolamine (25 species)
TG	Triacylglycerol (42 species)	PS	Phosphatidylserine (25 species)
dhCer	Dihydroceramide (12 species)	PI	Phosphatidylinositol (25 species)
Cer	Ceramide (12 species)	PG	Phosphatidylglycerol (25 species)
SM	Sphingomyelin (12 species)	BMP	Bis(Monoacylglycero)phosphate (25 species)
dhSM	Dihydrosphingomyelin (12 species)	AcylPG	Acyl phosphatidylglycerol (15 species)
MhSM	Monohydrosphingomyelin (12 species)	LPC	Lysophosphatidylcholine (9 species)
Sulf	Sulfatide (18 species)	LPCe	Ether lysophosphatidylcholine (9 species)
MhCer	Monohexosylceramide (24 species)	LPE	Lysophosphatidylethanolamine (9 species)
LacCer	Lactosylceramide (24 species)	LPEp	Plasmogen lysophosphatidylethanolamine (9 species)
GM3	Monosialodihexosylganglioside (18 species)	LPI	Lysophosphatidylinositol (9 species)
GB3	Globotriaosylceramide (12 species)	LPS	Lysophosphatidylserine (11 species)

Supplemental Figure 1. IFC images of gliosis makers in cortex. Representative images of IFC depict the staining patterns of DAPI, Iba1, GFAP, and Trem-2, respectively, in the cerebral cortex of mice aged 7 days, encompassing both saline-treated and n-3 treated groups. n=3-4. scale bar = 0.5 μm.

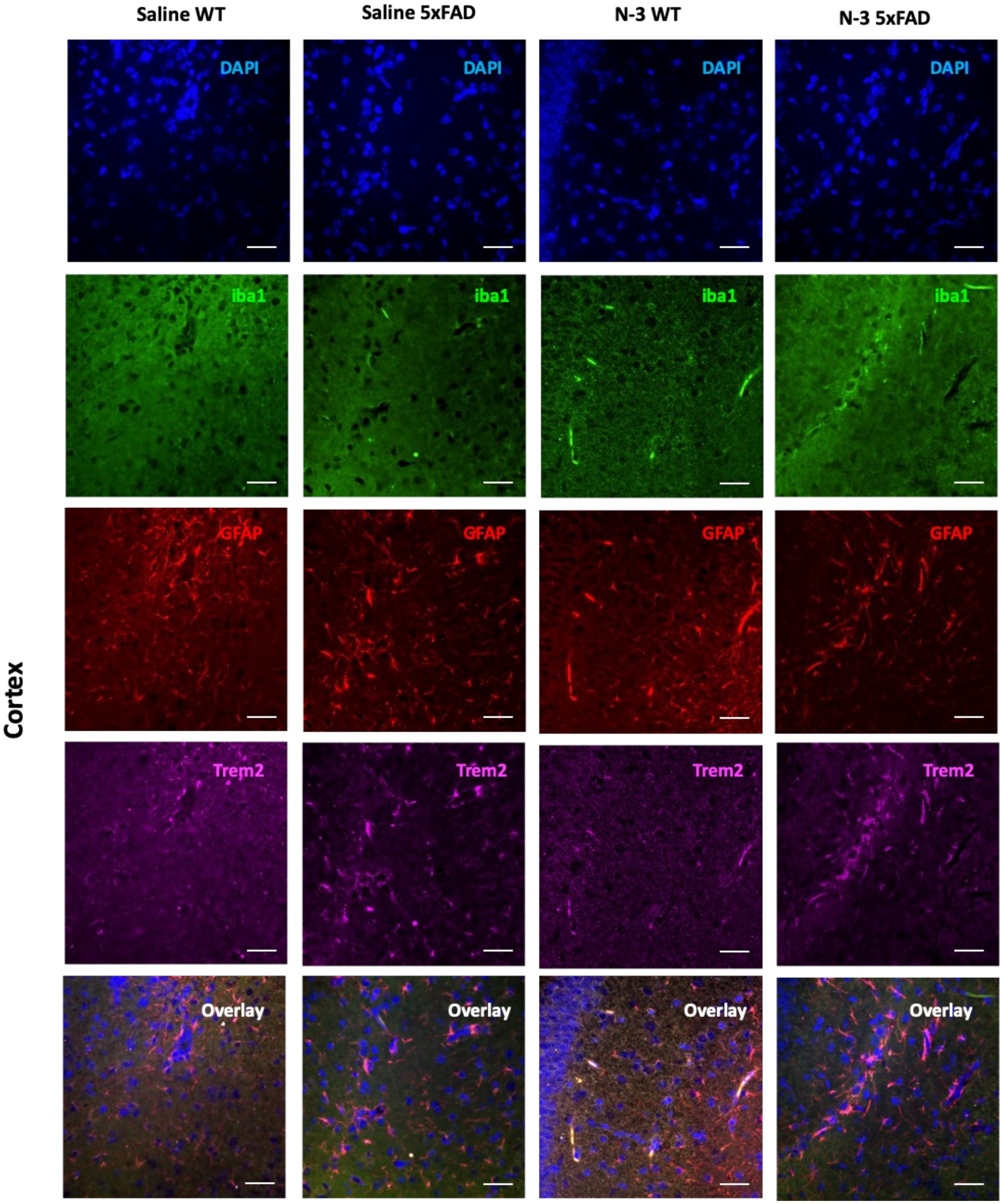

Supplemental Figure 2. IFC images of gliosis markers in adult cortex. Representative images of IFC depict the staining patterns of DAPI, Iba1, GFAP, and Trem-2, respectively, in the cerebral cortex of mice aged 5 months, encompassing both non-treated and n-3 treated groups. n=3-7. scale bar = 0.5 µm.

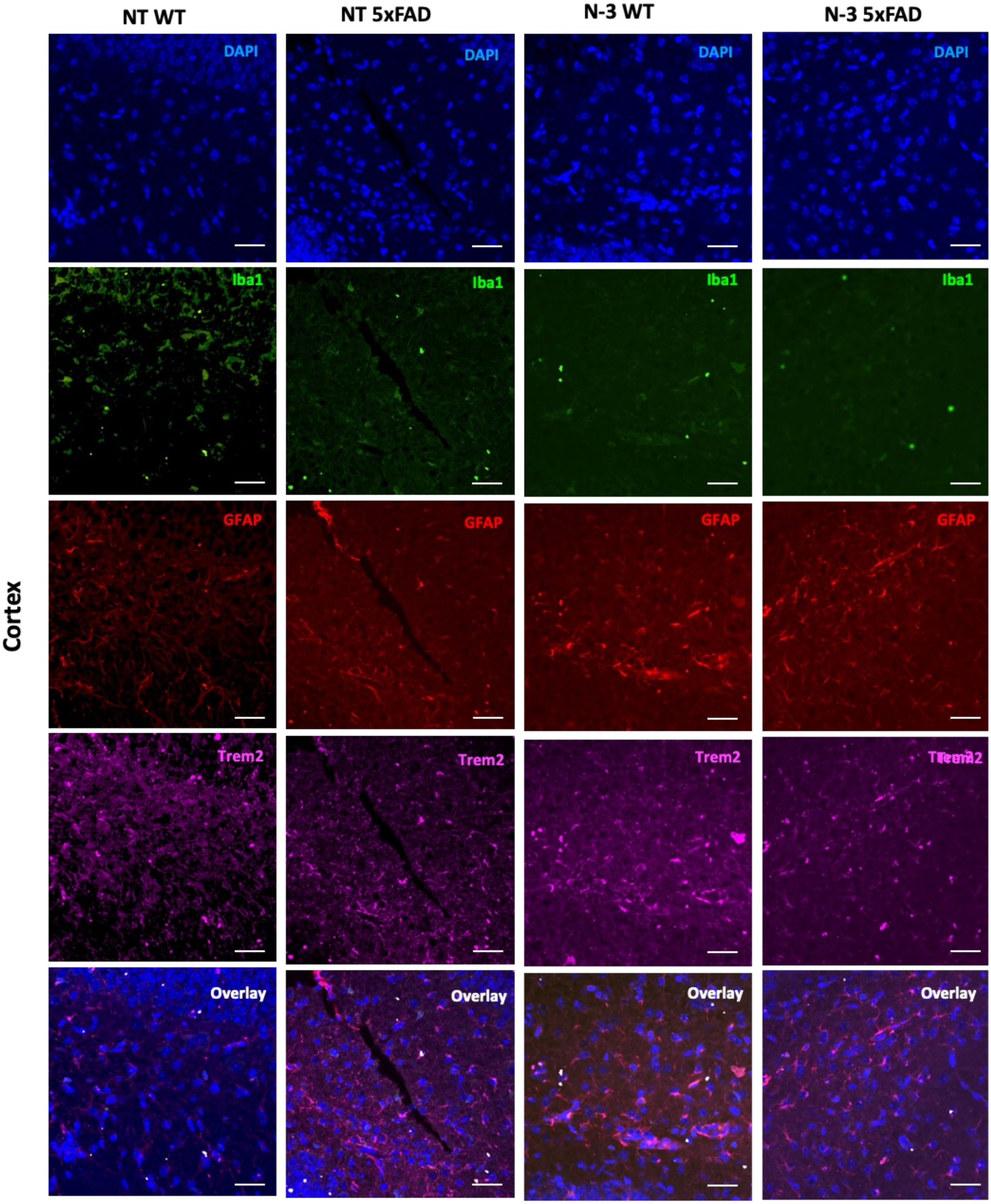

Supplemental Figure 3. Number of USV calls at postpartum day s 4, 6, 8, and 10 in WT and 5xFAD pups. (n: non-treated WT = 23, non-treated 5xFAD = 17, Saline WT = 7, Saline 5xFAD = 11, n-3 treated WT = 19, n-3 treated 5xFAD = 25). Two-way ANOVA, Sidak’s multiple comparisons test. Mean ± SEM.

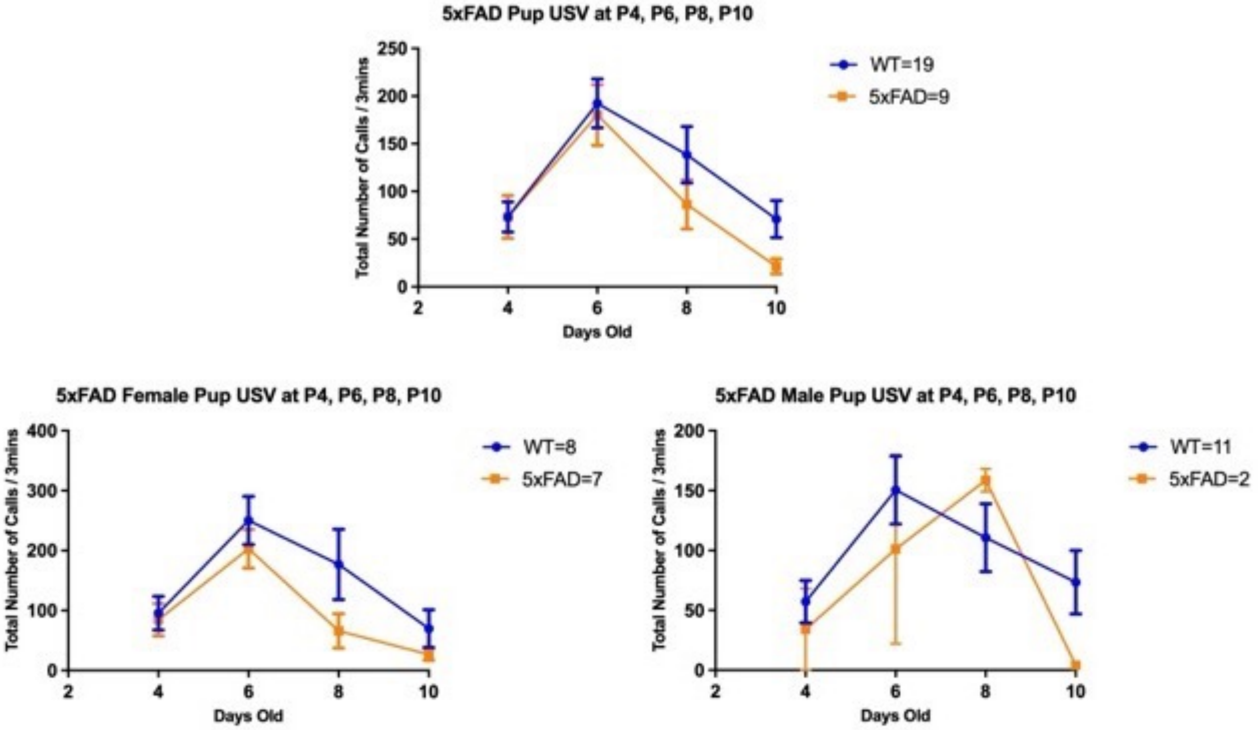

Supplemental Figure 4. Body weight of WT and 5xFAD pups at postpartum day 6. (n: non-treated WT = 41, non-treated 5xFAD = 28, Saline WT = 13, Saline 5xFAD = 15, n-3 treated WT = 22, n-3 treated 5xFAD = 28). Two-way ANOVA, Sidak's multiple comparisons test. Mean ± SEM. (*p < .05).

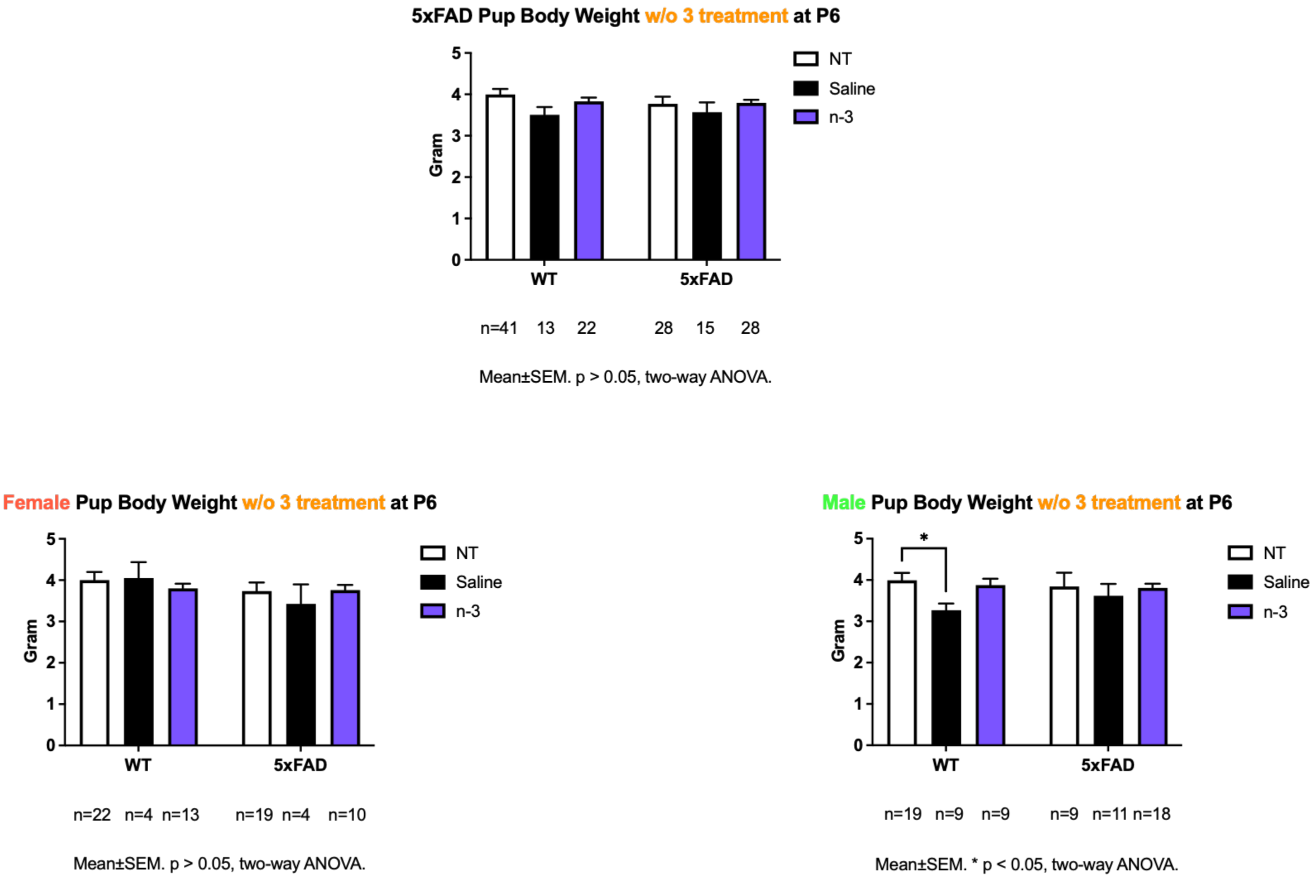

Supplemental Figure 5. Y-maze spontaneous alternation in 3-month-old F1 mice. (A) Percentage of spontaneous alternation and (B) total number of arm entries in 3-month-old F1 mice as determined by Y-maze as described in the Methods. (n=19). Mean ± SEM.

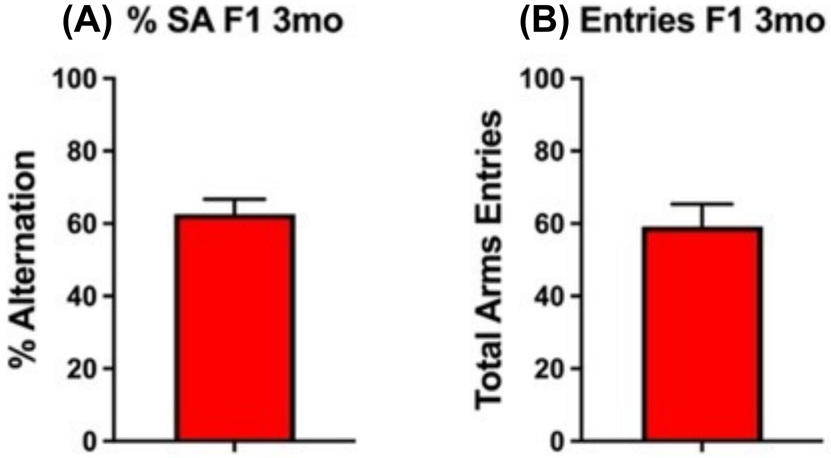

Supplemental Figure 6. N-3 effects on mouse cognitive and spatial memory capacities in 10-month-old mice. (A) Percent of exploration time in NT and n-3 WT and 5xFAD mice were recorded. (B) Total frequency of entrance to the novel arm in WT and 5xFAD mice with perinatal n-3 treatment. (NT WT = 13, NT 5xFAD = 15, n-3 WT = 8, n-3 5xFAD = 6). Two-way ANOVA, Sidak's multiple comparisons test. Mean ± SEM. *$p < 0.05$.

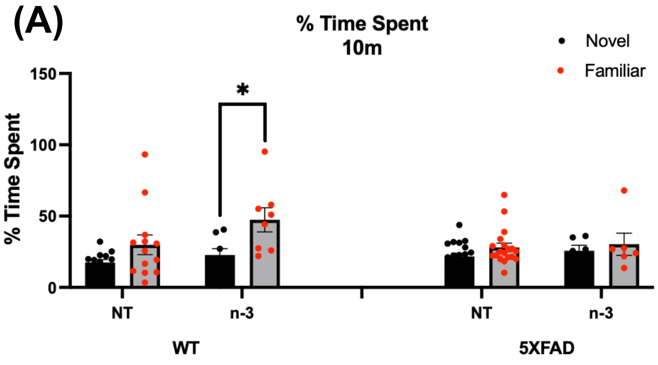

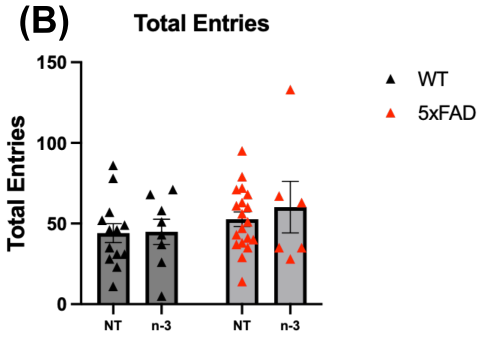

Supplemental Figure 7. Assessment for female and male 5.5-6-month-old NT mice in the open field (OF) maze. Distance traveled (A), vertical movement (B), and center time (C) across 40-minute time bins in 5.5-6-month-old 5xFAD and WT female mice (n=17-25). Distance traveled (D), vertical movement (E), and center time (F) across 40-minute time bins in 5.5-6-month-old 5xFAD and WT male mice (n=11-13). Unpaired T-test. Mean ± SEM. (*p < 0.05, **p < 0.01).

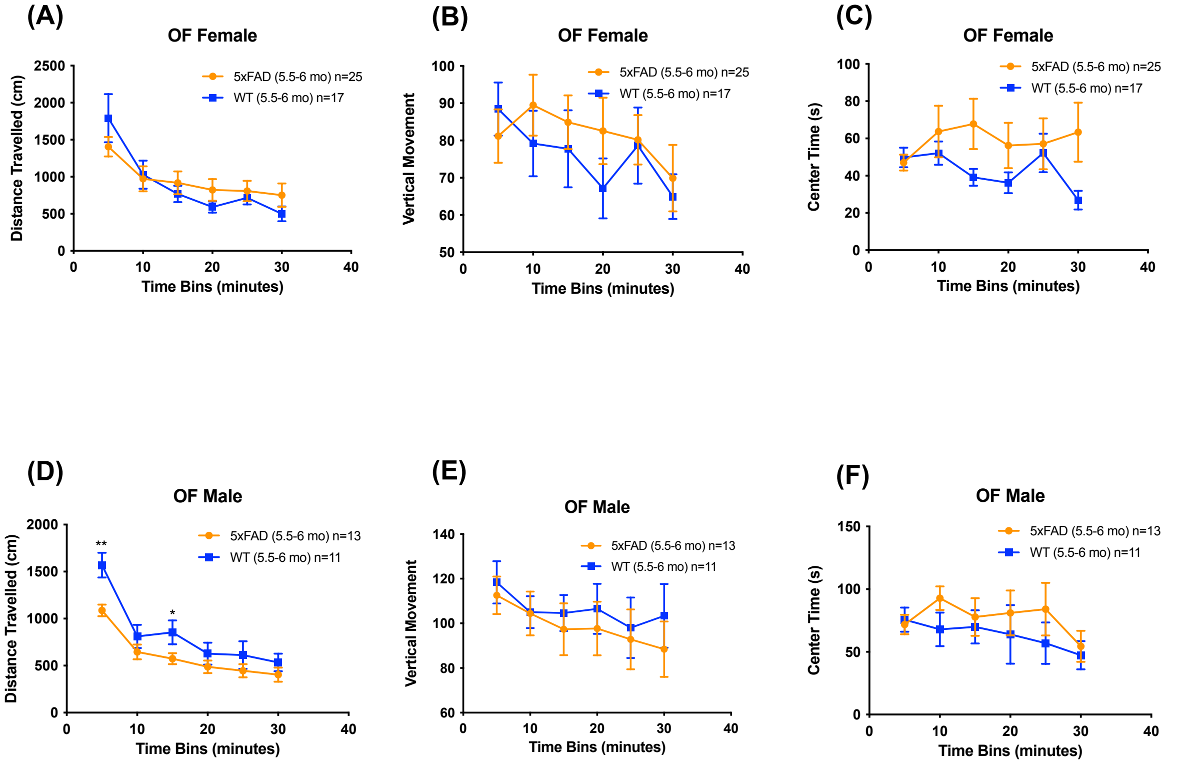

Supplemental Figure 8. Assessment for 10-month-old mice in the open field (OF) maze. Distance traveled (A), vertical movement (B), and center time (C) across 40-minute time bins in 10-month-old 5xFAD (n=16) and WT (n=19) mice. Unpaired T-test, Mean ± SEM. (*p < 0.05, **p < 0.01).

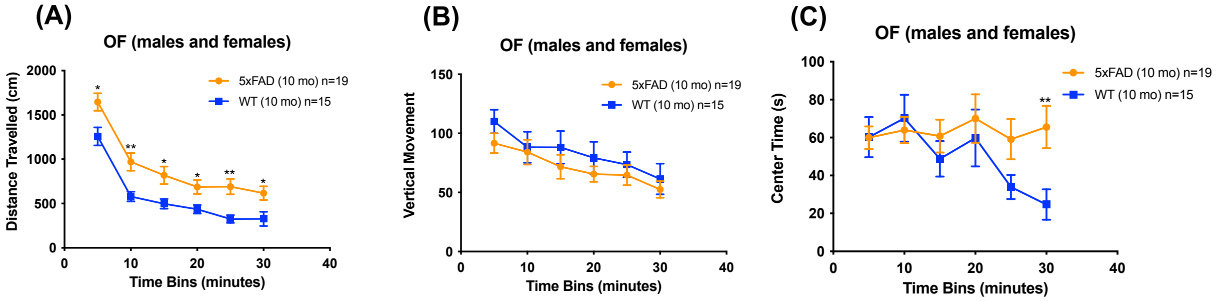

Supplemental Figure 9. N-3 treatments modulate CNS lipid composition. Lipidomic analysis was conducted on brain samples from WT and 5xFAD mice at P7 following perinatal saline or n-3 FA treatment. Lipid profiles were determined as described in the Methods. n = 3-4. Two-way ANOVA, Sidak's multiple comparisons test. Mean ± SEM. *p < 0.05, ** p < 0.01.

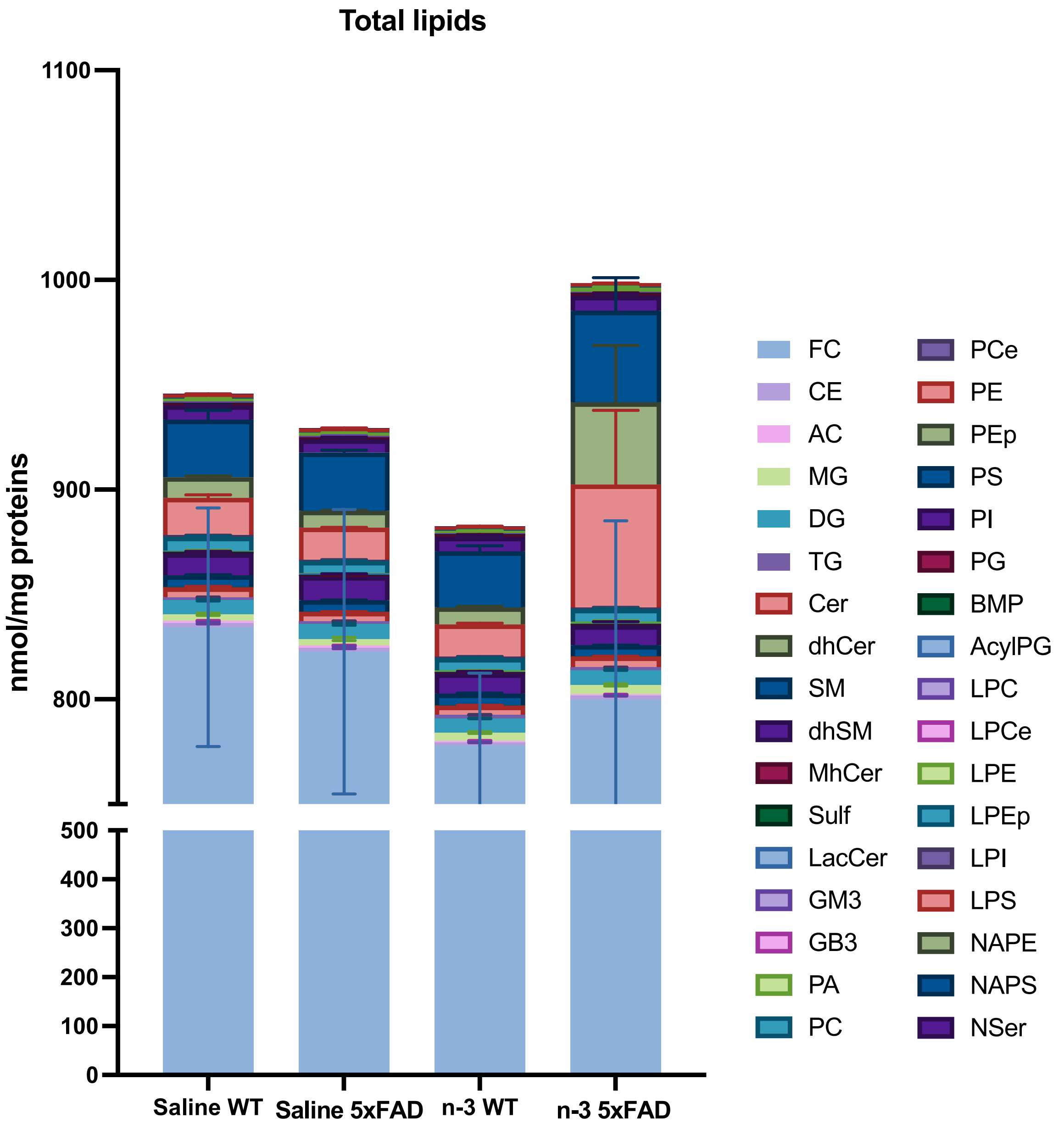

Supplement: Supplementary file 1 [file Data_Sheet_1.PDF]
